# Aggressive Stereotactic Radiosurgery Coupled With Immune and Targeted Therapy for Recurrent Melanoma Brain Metastases: A Case Report and Literature Review

**DOI:** 10.7759/cureus.26553

**Published:** 2022-07-04

**Authors:** Zhishuo Wei, Kaitlin Waite, Hansen Deng, Yana Najjar, Ajay Niranjan, L. Dade Lunsford

**Affiliations:** 1 Department of Neurological Surgery, University of Pittsburgh Medical Center, Pittsburgh, USA; 2 Department of Medicine, University of Pittsburgh Medical Center, Pittsburgh, USA

**Keywords:** cancer immunotherapy, brain tumors (primary or brain metastasis). neurologic complication of cancer or immunotherapy (cart), whole brain radiation, malignant melanoma metastasis, stereotactic radiosurgery srs

## Abstract

Melanoma is a complex disease with a high propensity for brain metastatic spread. Stereotactic radiosurgery (SRS) is a minimally invasive procedure to treat intracranial metastasis with a high rate of local tumor control. In this report, we describe the ongoing management of a patient with interval development of both new and recurrent brain metastases that required seven SRS procedures for a total of 48 brain metastases during a two-year interval while receiving concurrent immunotherapy with ipilimumab and nivolumab. The most recent imaging of the patient showed three brain areas of likely tumor progression despite maintenance nivolumab, and the treatment was recently changed to encorafenib and binimetinib. Combined management with immunotherapy, initial craniotomy, and repeated SRS for new brain metastases resulted in extended survival while preserving neurological function and reducing adverse treatment effects in a patient with advanced metastatic brain melanoma.

## Introduction

Melanoma is the fifth most prevalent cancer in the United States with an estimated onset of 106,110 new cases in 2021, accounting for 6% of the total cancer population [1]. Over one-third of patients with Stage IV melanoma eventually develop brain metastasis, which is associated with a historical median survival of four to six months [2-4]. With the advent of new systemic immunotherapy and targeted therapies, survivals in such advanced cases have now begun to improve [5].

Stereotactic radiosurgery (SRS) is an effective and non-invasive outpatient treatment modality that is now one of the most frequently used methods to control brain disease in melanoma patients with brain metastasis. Compared to traditional whole-brain radiation therapy (WBRT), SRS minimizes neurocognitive decline and improves local tumor control [6]. The clinical benefits of SRS performed in a single procedure exceed that of fractionated radiation therapy, so SRS has largely replaced the traditional indications for the utilization of WBRT [7,8].

In this report, we present the ongoing role of repeated SRS coupled with systemic immunotherapy in a melanoma patient with both new and recurrent brain metastases.

## Case presentation

A 30-year-old male presented with headache, right hemiparesis, and bilateral hand paresthesias. Computed tomography (CT) followed by magnetic resonance imaging (MRI) revealed a total of nine intracranial tumors, three of which were hemorrhagic, but no known primary source. Whole-body PET/CT did not show any other sites of disease. The patient subsequently underwent craniotomy with subtotal resection of the largest tumor located in the left frontal lobe, due to the proximity to the motor cortex. The neoplastic cells were stained after the excisional biopsy of the left frontal mass for MELAN A, SOX-10, and S100, supporting the diagnosis of melanoma. Pathology also revealed that metastatic melanoma was positive for BRAF V600E mutation and negative for NRAS and NF1. During recovery from the surgical procedure, intravenous dexamethasone (3 mg BiD over three weeks) was initiated temporarily and prophylactic levetiracetam (500 mg BiD over three months) was administered for seizure risk reduction due to post-operative mental status change and worsening cerebral edema.

**Figure 1:**
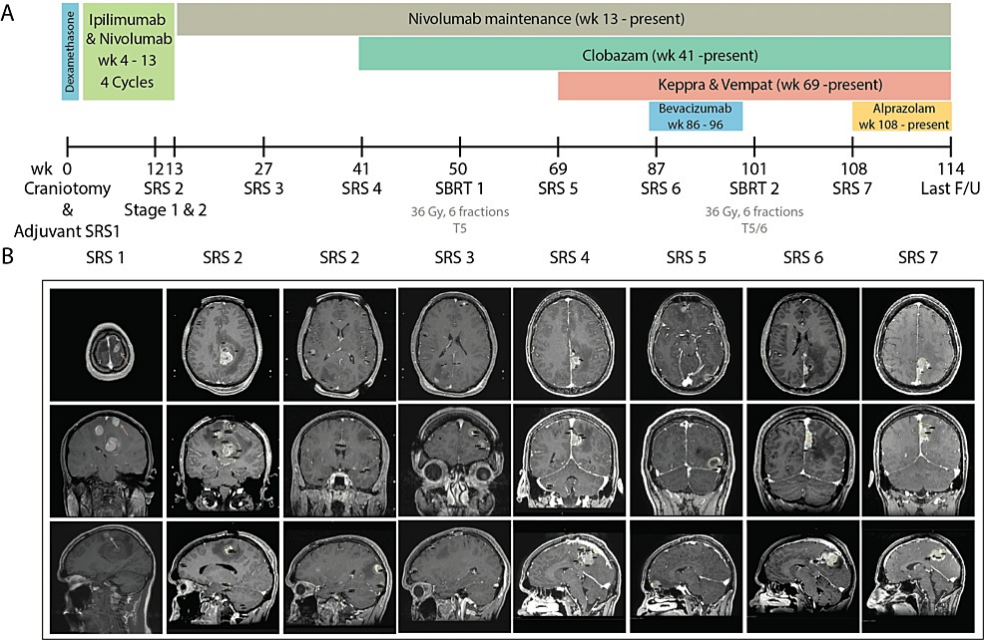
(A) Patient systematic disease treatment and radiosurgery history timeline. (B) Axial, sagittal and coronal views for pre-operative imaging and radiosurgery planning image for SRS 1-7. SRS: Stereotactic radiosurgery

Two days after craniotomy, the first SRS treatment targeted the residual tumor and resection cavity along with eight additional metastases (margin dose: 16-18 Gy to 50-90% isodose line). The patient experienced transient mental status changes that improved with temporary cerebrospinal fluid (CSF) drainage for two days. Systemic ipilimumab (3 mg/kg) and nivolumab (1 mg/kg) were then commenced for four cycles over two months one month after craniotomy and the patient was maintained on nivolumab (5.5 mg/kg) thereafter (Figure 1A).

The patient was followed up over a three-month interval during the first year after SRS. Decisions over tumor response and the development of new lesions were made in accordance with the response assessment in neuro-oncology brain metastases (RANO-BM) criteria. At the three-month follow-up, progressive disease was noted with 28 new metastatic lesions, while significant tumor responses were noted to the previously treated lesions and resection cavity. The patient underwent a second SRS 12 weeks after initial surgery, delivered in two separate procedures over two weeks apart (stage 1: 16 tumors, margin dose: 16 Gy, isodose 60-90%; stage 2: 12 tumors, margin dose: 16 Gy, isodose 60-90%).

Subsequently, the patient continued to have follow-up brain MRI scans at three-month intervals to monitor tumor response. This revealed a positive treatment response to treated lesions but again revealed new tumors at 27 (four tumors), 41 (three tumors), and 69 weeks (two tumors) respectively after the initial craniotomy. The patient underwent a third (four tumors, margin dose: 18 Gy, isodose 70-90%, week 27), fourth (three tumors, margin dose: 16 Gy, isodose 50-80%, week 41), and fifth SRS procedure (two tumors, margin dose: 16 Gy, isodose 50-60%, week 69) for these new metastases. The patient was placed on clobazam (5 mg BiD), and combined treatment of levetiracetam (1000 mg BiD) and lacosamide (50 mg BiD) for seizure control on weeks 41 and 69, respectively, and the patient was maintained on all three drugs until present (Figure 1).

**Figure 2:**
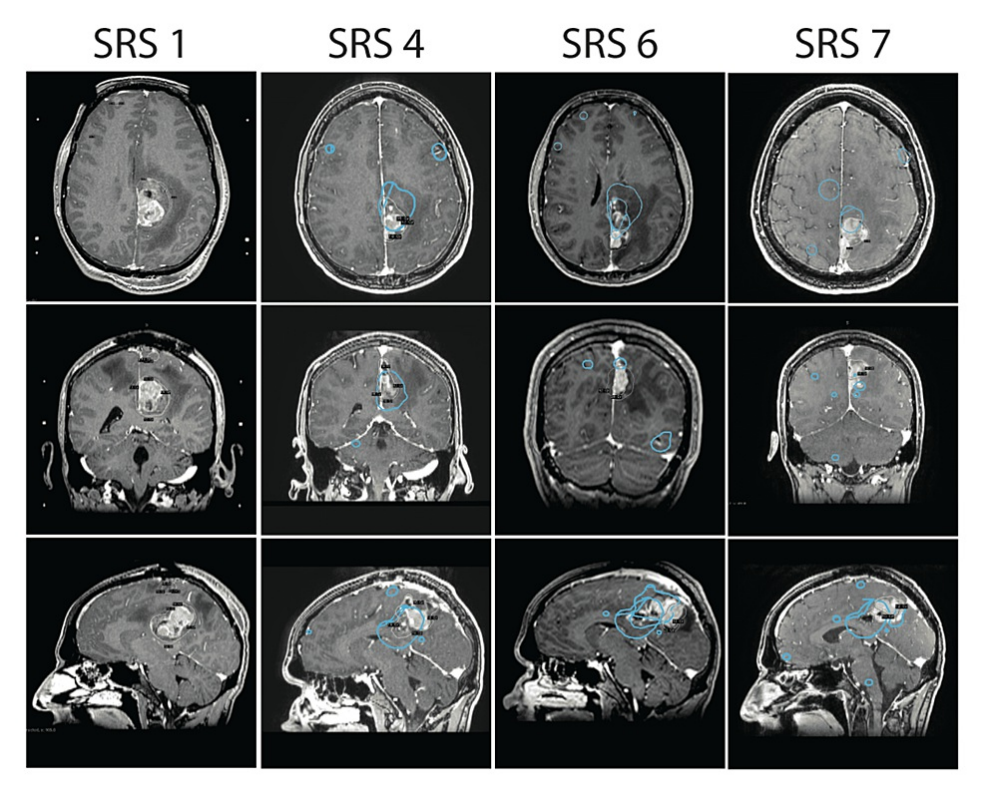
Local tumor recurrence in the motor cortex in axial, coronal and sagittal views with stereotactic dosage and location. Local recurrence occurred within 41 weeks between SRS 1 and 4, 46 weeks between SRS 4 and 6, and 21 weeks between SRS 6 and 7. SRS: Stereotactic radiosurgery

**Figure 3:**
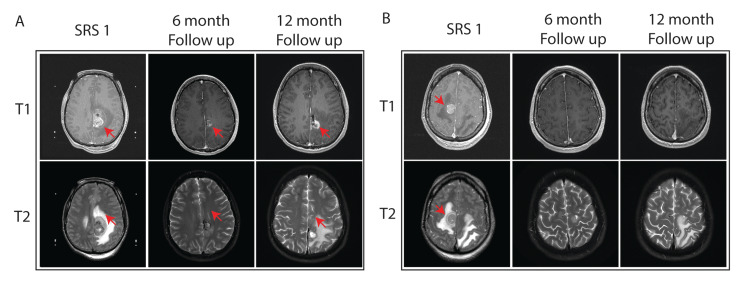
Peritumoral edema response to bevacizumab at 6 and 12 months after SRS SRS: Stereotactic radiosurgery

Eighty-seven weeks after the initial craniotomy, the patient represented partial seizures associated with intermittent right lower extremity cramping and stiffness. His MRI revealed progression of the left parafalcine mass now associated peritumoral edema. The patient underwent a sixth and seventh SRS for locally progressive tumors at 87 and 108 weeks after his initial craniotomy, and was placed on alprazolam (0.5 mg PRN) at week 108 for further seizure control. Intravenous bevacizumab at three-week intervals was initiated at 86 weeks to reduce surrounding edema while avoiding systemic corticosteroid administration (Figures 2, 3; Table 1).

**Table 1:** Tumor characters and treatment parameters for each SRS SRS: Stereotactic radiosurgery

Number of SRS	Number of lesions	Tumor cumulative volume (cc)	Margin dose (Gy)	Isodose (%)	Tumor in eloquent area?
1	9	33.1	16-18	50-90	Y
2 – Stage 1	16	5.467	16	60-90	N
2 – Stage 2	12	3.128	16	60-90	N
3	4	0.356	18	70-90	N
4	3	10.698	16	50-80	Y
5	2	4.66	16	50-60	N
6	1	3.252	20	55	Y
7	1	4.646	16	55	Y

The patient has been maintained on nivolumab to control his systemic disease and anticonvulsant medication to prevent partial seizures. At the most recent follow-up, 114 weeks after the initial craniotomy, additional tumor progression was confirmed for three previously treated tumors. There was also evidence of systemic progression of the disease, with enlargement of lesions at T5 and the left iliac crest. Systemic therapy was recently changed to encorafenib (75 mg QD) and binimetinib (45 mg BiD). This was chosen instead of dabrafenib/trametinib as the patient has had a seizure with fever, and the toxicity of fevers is more common with dabrafenib/trametinib. The patient continues with clinical and imaging evaluations at three-month intervals. Additional radiosurgery is anticipated if additional symptoms evolve. During this two-year survival, the patient has continued full-time employment with minimal symptom burden, maintaining an ECOG (eastern cooperative oncology group) performance status of 0-1.

## Discussion

SRS is an important, minimally invasive strategy for patients with newly diagnosed or recurrent metastatic cancer to the brain, with local brain tumor control rates at one year after SRS that vary between 80 to 95% [9-11]. Compared to conventional WBRT, SRS results in improved local tumor control, long-term survival, and neurocognition preservation in patients with metastatic melanoma [12]. In a prospective study including 213 participants with one to three brain metastases, Brown et al. [13] reported that patients treated with SRS alone showed reduced cognitive deterioration compared to patients who had combined SRS and WBRT.

Both the number and the cumulative tumor volume must be evaluated in a patient when determining the course of treatment. Recent evidence confirms that cumulative tumor volume is an important prognostic factor in determining patient survival [14,15]. In the present case, we found that repeat SRS after initial resection of a symptomatic tumor was effective in the treatment of multiple new and recurrent brain melanoma metastases (Figure 2). Serizawa et al. [16] reported no difference in survival between patients with 2-4 and 5-10 breast, lung, and gastrointestinal cancer brain metastases after retrospectively reviewing 1,508 patients, allowing SRS to treat 10 or more lesions at a time with no cases of carcinomatous meningitis [9,17]. In a prospective study of 1194 patients, Yamamoto et al. [14] reported that SRS without WBRT is non-inferior in treating patients with 5-10 brain metastasis compared to patients with 2-4 brain metastasis. Taken together, the number of brain metastases may not be the strongest risk factor in predicting patient survival.

SRS minimizes radiation delivery to the surrounding brain and reduces neurologic deficits after treatment, especially in critical locations. In a retrospective study of 547 patients with metastases located in the brain stem, Redmond et al. [11] reported an 85-95% one-year local tumor control rate with a higher tumor control rate correlated with higher-margin doses. Luther et al. [18] retrospectively reviewed 96 patients with brain metastasis located in the motor cortex, and reported that 31% of patients had improved motor function and 50% of patients had a stable motor function after SRS. In the present case, we showed that tumors in the left and right motor cortex and pons were successfully controlled with single or repeated SRS (Table 2).

**Table 2:** Treatment parameters for tumors in neurologically critical areas SRS: Stereotactic radiosurgery

Tumor location	Cumulative tumor volume (cc)	SRS 1 Margin dose @isodose	SRS 2 Margin dose @isodose	SRS 3 Margin dose @isodose	SRS 4 Margin dose @isodose	Tumor response at the last F/U
L motor cortex	25.638	16 Gy @ 50%	16 Gy @ 60%	20 Gy @ 55%	16 Gy @ 55%	Regressed
R motor cortex	3.577	16 Gy @ 60%	n/a	n/a	n/a	Disappeared
Brain stem	0.033	18 Gy @ 85%	n/a	n/a	n/a	Disappeared

In this case of metastatic melanoma with central nervous system (CNS) involvement, multi-modality management was key to ensuring extended survival while maintaining a high quality of life. Concurrent systemic therapy in this patient included ipilimumab and nivolumab, which is the standard of care for patients with melanoma brain metastases [5,19,20]. The patient underwent a total of seven repeated SRS procedures for a total of 48 new or locally progressive melanoma metastases. Three of these tumors were located in brain regions with critical neurological function. During this two-year interval, oral anticonvulsants provided seizure control for tumors in epileptogenic brain regions. A 10-week course of intravenous bevacizumab was used to treat peritumoral edema while avoiding corticosteroid administration. Edema surrounding the tumors in the left and right motor cortex improved significantly 12 months after the first SRS (Figure 3). Aggressive intermittent repeat SRS coupled with systemic immune and targeted therapy can result in prolonged survival in patients with intracranial melanoma metastases.

## Conclusions

Following initial partial resection of the first metastatic tumor, this patient required subsequent multimodality management with systemic immune and targeted therapy coupled with repeated SRS procedures to control new or progressive intracranial disease. Combined management with immunotherapy, initial craniotomy, and repeated SRS for new brain metastases resulted in extended survival while preserving neurological function and reducing adverse treatment effects in a patient with advanced metastatic brain melanoma.
